# Agronomic impacts of chemically and microbiologically characterized compost tea in Mediterranean volcanic soils

**DOI:** 10.3389/fpls.2025.1524884

**Published:** 2025-02-28

**Authors:** Giuliano Bonanomi, Ayoub Idbella, Giandomenico Amoroso, Giuseppina Iacomino, Mara Gherardelli, Andrea De Sio, Franco Saccocci, Ahmed M. Abd-ElGawad, Mauro Moreno, Mohamed Idbella

**Affiliations:** ^1^ Department of Agricultural Sciences, University of Naples Federico II, Portici, NA, Italy; ^2^ Task Force on Microbiome Studies, University of Naples Federico II, Naples, Italy; ^3^ Laboratory of Organic Synthesis, Extraction, and Valorization, Faculty of Sciences Ain Chock, Hassan II University, Casablanca, Morocco; ^4^ Professional Agronomist, Naples, Italy; ^5^ Plant Production Department, College of Food & Agriculture Sciences, King Saud University, Riyadh, Saudi Arabia; ^6^ AgroBioSciences (AgBS) Program, College of Agriculture and Environmental Sciences, Mohammed VI Polytechnic University, Ben Guerir, Morocco

**Keywords:** compost tea, C/N ratio, microbiome, organic amendment, plant biostimulation, plant pathogens

## Abstract

Compost tea is widely recognized for its beneficial effects on crop growth and soil health. However, its efficacy varies depending on the composition of the feedstock and brewing conditions. This study investigates the chemical composition and agronomic impact of compost tea prepared from a commercial mixture of plant residues and animal manure. Standard chemical analyses, combined with solid-state ^13^C CPMAS NMR spectroscopy, were employed to characterize the organic chemistry of the feedstock. High-throughput sequencing of bacterial and eukaryotic rRNA gene markers was used to profile the microbiota. Compost tea was applied to three crops, *Allium cepa*, *Beta vulgaris*, and *Lactuca sativa*, grown in protected Mediterranean environments on volcanic soils. The ^13^C CPMAS NMR analysis revealed that the feedstock is predominantly composed of plant-derived tissues, including grass straw, nitrogen-fixing hay, and animal manure, with a significant presence of O-alkyl-C and di-O-alkyl-C regions typical of sugars and polysaccharides. Additionally, the chemical profile indicated the presence of an aliphatic fraction (alkyl-C), characteristic of lipids such as waxes and cutins. The compost tea microbiome was dominated by *Pseudomonadota*, with *Pseudomonas*, *Massilia*, and *Sphingomonas* being the most prevalent genera. Compost tea application resulted in significant yield increases, ranging from +21% for lettuce to +58% for onion and +110% for chard. Furthermore, compost tea application reduced slug damage and enhanced the shelf life of lettuce. These findings highlight the bio-stimulant potential of this standardized compost tea mixture across different vegetable crops.

## 1 Introduction

The soil microbiome plays a critical role in agroecosystems (Hartmann and Six, 2023; Benmrid et al., 2024). It facilitates organic matter decomposition, driving nutrient mineralization (Sokol et al., 2022), interacts with both organic and mineral soil fractions to form aggregates (Wilpiszeski et al., 2019), and competes for resources, thereby limiting pathogen proliferation (Schlatter et al., 2017). However, certain agronomic practices in intensive agriculture can diminish microbiome diversity and functionality (Idbella and Bonanomi, 2023). For instance, excessive mineral fertilization creates an ionic “rain” that can lead to soil salinization and abrupt pH fluctuations (Zhang et al., 2022). The progressive decline in soil organic carbon content also reduces enzymatic activity and microbial biomass (Bonanomi et al., 2011), while frequent pesticide and fumigant applications cause negative selection pressures on fungi and bacteria in both the phyllosphere and rhizosphere (Cesarano et al., 2017).

Various strategies have been developed to recover and promote the growth of a healthy microbiome, including the use of beneficial microbial inoculants and the application of organic amendments. Among the most commonly used biostimulant microorganisms are fungi from the *Trichoderma* genus and bacteria from the *Bacillus*, *Pseudomonas*, and *Streptomyces* genera, among others (Raymaekers et al., 2020). These beneficial microbes exert their effects through multiple mechanisms, such as antibiotic production, the synthesis of compounds with auxin-like activity (Tzipilevich et al., 2021), competition in the rhizosphere (Chepsergon and Moleleki, 2023), and mycoparasitic activity (Ghorbanpour et al., 2018), thereby combining both biocontrol and bio-stimulatory properties (Khan et al., 2023). Despite numerous studies, the effectiveness of beneficial microorganisms often falls short of synthetic products, largely due to complex interactions with environmental conditions that hinder their establishment and spread (Bellini et al., 2023). In this context, some studies have explored combining beneficial microorganisms with organic amendments to provide a suitable organic substrate that enhances microbial establishment and proliferation (Bonanomi et al., 2022; Chouyia et al., 2022). For instance, a recent study demonstrated that combining the fungus *Trichoderma harzianum* with different types of biochar resulted in greater effectiveness compared to using the individual products alone (Iacomino et al., 2023).

The application of organic amendments is one of the most commonly used traditional techniques to restore organic carbon content, enhance soil fertility, and modulate the soil microbiome (Idbella et al., 2024). The effectiveness of this technique is primarily influenced by factors such as the nature of the organic feedstocks (e.g., compost, biochar, peat), their stage of decomposition, and the quantities applied, which can range from less than 0.5 to over 100 t ha⁻¹ year⁻¹ (Bonanomi et al., 2007). Long-term field trials (Diacono and Montemurro, 2011) have primarily focused on evaluating the immediate biological and agronomic effects of organic amendments, typically applying them once at the start of the experiment (Bastida et al., 2008; Ferreras et al., 2006). This approach mirrors common agricultural practices, where organic amendments are added once or twice a year. However, applying large quantities of organic amendments in a single application leads to rapid microbial growth followed by a sharp decline in microbial biomass, causing fluctuations in the microbiome and its functionality (Acea and Carballas, 1999). Unfortunately, few studies have explored the relationship between soil functioning and the frequency of organic amendments. Some research has investigated the impact on soil fungistasis (Bonanomi et al., 2017), enzymatic activity (Stark et al., 2008), soil respiration (Nett et al., 2012), and nitrogen (N) mineralization (Duong et al., 2009). Previous studies have shown that soils treated with multiple applications of organic amendments exhibit larger, more active microbial biomass and increased enzymatic activities compared to single applications (Dick et al., 1988; Pilla et al., 2023).

In this context, the use of compost tea offers a promising strategy that combines the benefits of beneficial microbes with organic amendments. Compost teas, which are produced by short-term aerobic or anaerobic brewing of undecomposed or decomposed solid organic feedstocks in water, have long been recognized for their positive effects on crop and soil health (Scheuerell and Mahaffee, 2002). However, the effectiveness of compost tea is highly variable and depends on factors such as the composition of the feedstock and the brewing conditions, including feedstock concentration, oxygen availability, and brewing duration. Developed several decades ago, compost tea is now primarily used in organic farming as a biostimulant and for pathogen control (Hartmann and Six, 2023). In recent years, interest in compost tea has resurged due to rising fertilizer costs and the decreasing availability of synthetic active substances for disease management. However, challenges in standardizing the product and the necessity of producing it fresh on the farm, just before application, have limited its widespread adoption. Optimizing and standardizing the production process, based on the chemical properties of the initial feedstock and conditions such as oxygen levels, temperature, and brewing duration, are crucial steps toward making compost tea a viable and reliable alternative.

In this study, we investigated the chemical and microbiological composition, as well as the agronomic impact, of a compost tea made from a commercial mixture of plant residues and animal manure. The first hypothesis states that it is possible to produce compost tea with a relatively stable microbiome if the quality of the starting organic feedstock and the process conditions in terms of oxygenation are controlled. Furthermore, the second hypothesis states that such compost tea has biostimulatory effects on crop growth with suppressive capabilities against some pathogens and pests. In order to test these hypotheses, we combined solid-state ^13^C CPMAS NMR to analyze the chemical properties of the source organic feedstock, while high-throughput sequencing of bacterial 16S rRNA gene markers was employed to characterize the microbiota. Compost tea was then applied to three crops (*Allium cepa*, *Beta vulgaris*, and *Lactuca sativa*) over four cultivation cycles in protected Mediterranean environments on volcanic Vitric Andosols. Crop yield, phytosanitary status, and, in the case of lettuce, shelf life were evaluated. The main objectives of the study were:

1. To chemically characterize the initial feedstock and the compost tea at the end of the production process.
2. To describe the bacterial microbiome of compost tea.
3. To quantify the impact of compost tea on the production and phytosanitary status of three horticultural crops.

## 2 Materials and methods

### 2.1 Solid feedstock chemistry and human pathogen assessment

The studied amendant is a commercial compost tea named Stimol-C^®^ and produced by the company GWA-GimaWater & Air S.r.l. The organic feedstock consists of mixed plant materials, straw and composted cow manure from biological farms. The following properties of the solid fraction, before aerobic brewing, were characterized: water content, pH, organic carbon, humic and fulvic acids content, total nitrogen, C/N ratio, and electrical conductivity (EC). In detail, Water content was determined gravimetrically following oven dehydration at 105°C for 5 days. EC and pH were determined in water extract (1:10 w/v compost: water) with pH and EC meters (HANNA instruments, model HI5521). Organic carbon and total nitrogen were determined in microsamples (10 mg each) using an elemental analyzer (NA 1500, Carlo Erba Strumentazione, Milan, Italy). The C/N ratio was calculated from the values of organic carbon and total nitrogen. Finally, humic and fulvic acids were determined by extraction using a combination of sodium hydroxide and sodium pyrophosphate solution (Spaccini et al., 2006).

The presence of human pathogens *Escherichia coli* and *Salmonella* spp. has been assessed on organic feedstock following the official methods defined by the Italian Ministry of Agricultural Food and Forestry Policies (MIPAF 2014).

The organic fraction has also been characterized by the high-throughput method of ^13^C cross-polarized magic angle spinning NMR spectroscopy (^13^C CPMAS NMR) that offers several advantages over other techniques (Kögel-Knabner, 2002). First of all, ^13^C CPMAS NMR is performed in a solid state using directly the raw, untreated organic substrate. This allows for a direct characterization without the bias introduced by different extraction methods such as NaOH with or other organic solvents (Lehmann and Kleber, 2015). Second, ^13^C CPMAS NMR allows the description of several important chemical characteristics of organic substrate, and these data can be analyzed and associated with ecosystem functions. Briefly, the ^13^C CPAMS NMR analyses were performed under the same experiment conditions at the CERMANU Interdepartmental Research Centre (University of Napoli Federico II). The NMR spectra were recorded by a spectrometer Bruker AV 300 (Bruker Instrumental Inc., Billerica, MA, USA) that was equipped with a wide bore of 4 mm magic angle spinning (MAS) probe. The experimental set up layout consisted of MAS with 13,000 Hz of rotor spin rate 2 second of recycle time, 1 ms of contact time, 30 ms of acquisition time and 4000 scans, an orbital period of 1 second and a contact time of 1 ms with an acquisition time of 20 ms and 2000 scans (for further details see Bonanomi et al., 2013). Spectral ranges with the corresponding classes of carbon type, were selected in agreement with previous publications (review in Kögel-Knabner, 2002). In detail, the following chemical shift regions that represent major carbon types were studied: 0 to 45 ppm = alkyl + alpha-amino C; 46 to 60 ppm = N-alkyl C (56 ppm = methoxyl, alpha-amino); 61 to 90 ppm = O-alkyl C; 91 to 110 ppm = di-O-alkyl C (103–105 ppm = anomeric C in carbo-hydrates, quaternary aromatic carbons in tannins); 111 to 140 ppm = H- and C-substituted aromatic C (126 ppm = unsubstituted); 141 to 160 ppm = O-substituted aromatic C (phenolic and O-aryl C, 147–153 ppm = heterosubstituted, vanillyl plus syringil lignin units); and 161 to 190 ppm = carbonyl C (172 ppm = carboxyl + amide, 198 ppm = ketone/aldehyde). The relative contribution of the different regions was determined by integrating using MNova NMR software (Mestre-lab Research), with data expressed as a percentage of the total area.

### 2.2 Compost tea preparation, chemistry and microbiome

An aerated compost tea was prepared by mixing tap water with the organic feedstock in a ratio of 2:100 (w/v), in a polyethylene non degradable 200 L container. The solid fraction was contained in a porous bag with a mesh of 0.2 mm and thus immersed in the water for 48 hours of brewing. During the process the liquid was aerated with an air pump (Secoh Air Pump – model JDK-40) that introduced 40 L of air per minute and generate fine air bubbles. At the end of the 48 hours of brewing, the compost tea was applied directly to the plant species for the agronomic tests without storage phases. Compost tea was produced at weekly intervals and applied to crops immediately after brewing.

The compost tea was chemically characterized by the following parameters: pH, electrical conductivity, dissolved organic carbon (DOC), dissolved organic nitrogen (DON), biological oxygen demand (BOD5), dissolved oxygen, nitrate and ammonium. Dissolved oxygen, EC and pH were assessed with a multi-parametric probe (M40+ instrument, Crison). DOC and BOD5 were assessed, respectively, by the IRSA-CNR 5110 protocol and with the respirometric method by means of the Oxitop OC100 system (OXITO-C). Nitrate and ammonium were determined by a DR 3900 Spectrophotometer (Hach, Loveland, CO, USA) using the manufacturer kits LCK 340 for nitrate (assay range 5–35 mg L^-1^) and LCK 303 for ammonium (assay range 2–47 mg L^-1^). Iron (Fe) was determined by ICP-MS. While sulphates and phosphates were measured by ion chromatography (Dionex™, Thermo Fisher Scientific). The phytotoxicity bioassay was carried out with *Lepidium sativum*, a species recognized as very rapid and sensitive to phytotoxins and therefore used in official tests to certify quality compost (Barral and Paradelo, 2011). The test was conducted according to Zucconi et al (Zucconi, 1981). and the results were expressed as a germination index that takes into account both the percentage of germination and root elongation.

The microbiota of compost tea was characterized by four independent brewing processes. For each brewing process, three replicate DNA extractions were performed, and the resulting extracts were pooled to create one composite extraction per process, resulting in a total of four composite DNA samples. Briefly, DNA extraction from the compost tea samples was carried out using the DNeasy PowerSoil Pro Kit (Qiagen), with adaptations to suit liquid samples. Specifically, 10 mL of compost tea was centrifuged at 10,000 × g for 10 minutes to pellet microbial cells. The supernatant was discarded, and the resulting pellet was resuspended in the lysis buffer provided in the kit. The remaining steps of the extraction followed the manufacturer’s protocol for soil samples. The extracted DNA was quantified using a NanoDrop spectrophotometer. PCR and sequencing followed the protocols of (Idbella et al., 2022). Specifically, the V3–V4 regions of bacterial 16S rRNA were amplified using primers from (Berni Canani et al., 2017). PCR conditions included an initial denaturation at 95°C for 3 minutes, followed by 35 cycles of denaturation (95°C for 30s), annealing (55°C for 30s), and extension (72°C for 45s), ending with a final extension at 72°C for 10 minutes. Amplicons were sequenced using the Illumina MiSeq platform, adhering to the Illumina metagenomic workflow. The resulting reads were processed using the DADA2 package in R software (version 4.4.2). First, quality filtering was performed, with reads truncated at 240 bp for both forward and reverse reads to remove low-quality base pairs. Primer sequences were removed using the *filterAndTrim* function and reads with ambiguous bases (N) or a maximum expected error rate (maxEE) >2 were discarded. Paired-end reads were merged using the *mergePairs* function, allowing for a minimum overlap of 12 bp. Chimeric sequences were identified and removed using the *removeBimeraDenovo* function in the consensus mode. Amplicon sequence variants (ASVs) were then inferred using DADA2’s error model, trained on the dataset using the *learnErrors* function. Taxonomic classification was performed by comparing ASVs against the SILVA database (release 138) using the *assignTaxonomy* function with a minimum bootstrap confidence threshold of 80%.

### 2.3 Greenhouse experiments

To test the performance of compost tea on plant growth, an experiment was conducted in a plastic tunnel of the Department of Agriculture using horticultural plants. The soil is a Silandic and Vitric Andosols according to USDA Soil Taxonomy System (Soil Survey Staff 1999). The soil is sandy with 79.3% sand, 19.4% silt and 1.3% clay, 1.44% organic carbon content, pH of 7.12, EC of 0.26 dSm^-1^, NH_4_ (ammonium) concentration of 0.15 mg kg^-1^, total nitrogen (N) content of 16.8 g kg^-1^, C/N ratio of 6.8, phosphorus pentoxide (P_2_O_5_) content of 107.5 mg kg^-1^, total limestone of 6.79 g kg^-1^, cation exchange capacity of 14.4 meq 100 g^-1^, exchangeable Calcium of 10.1 meq 100 g^-1^, ex-changeable Magnesium of 1.83 meq 100 g^-1^, exchangeable Sodium of 0.14 meq 100 g^-1^, exchangeable Potassium of 1.37 meq 100 g^-1^, available Iron of 13.2 mg kg^-1^, available Copper of 12.0 mg kg^-1^, available Zinc of 10.9 mg kg^-1^, and available Manganese of 4.11 mg kg^-1^.

Concerning greenhouse microclimate, the OFYLIA 4.0-C sensor has monitored multiple environmental parameters. It has recorded a temperature range of 14°C to 35°C and a daylight intensity of 1250 µmol/s/m², representing the average value obtained from measurements taken at 12:00 p.m. over five sunny days. Additionally, the sensor measures soil moisture, with reported values ranging from 13% to 25%, influenced by the irrigation process.

The experimental tunnel, having an area of approximately 500 m^2^ was divided into 15 plots (4 m long and 1 m wide each) to obtain a randomized block design with five field replicates for the untreated control, the compost tea applied at full concentration (2/100, w/v) identified as compost tea 2%, and diluted ten times with water and indicated as compost tea 1%. Three vegetable species were tested: lettuce (*Lactuca sativa*, cv imanis), Onion (*Allium cepa*, cv tropea rossa lunga), chard (*Beta vulgaris*, cv cicla). Overall, four crop cycles were conducted (i.e., one for chard, two for lettuce and one for onion). The lettuce cycles lasted 60 days, the chard cycle 60 days and the onion cycle 128 days. During the chard cycle, three leaf cuts were carried out. Compost tea was applied as foliar spray every seven days in equivalent quantities of 1600 ml m^-2^, while the control was treated with the same amount of water. A total of eighteen independent brewing processes were conducted for weekly applications of compost tea to the different crops. The compost tea was always applied in the morning between 9.00 and 10.00 a.m. using an electric backpack pump (Stocker model with 15 l tank and 5 bar operating pressure). To represent and simulate the typical agronomic practices currently in use in the area, at the beginning of the experiment all plots were fertilized including with the application of a ternary fertilizer Nitrophoska^®^ Special 12-12-17, in dosage of N (60 kg ha^-1^); P (60 kg ha^-1^) and K (85 kg ha^-1^). Finally, experiments were conducted without the use of pesticides to evaluate the impact of compost tea on pathogens and pests of different species.

Total crop yields were recorded at the end of each cycle to quantify the amount of commercial production in all experimental plots. At the end of the cycle, the attacks of parasites and pathogens were evaluated using a scale from 0 to 5 with the lowest values indicating no attack and the highest values an attack of high intensity. In detail, for lettuce, the attack of slugs was evaluated while for beet, of cercospora leaf spot (*Cercospora beticola*), of manganese deficiency and chewing insects. Finally, for lettuce, shelf life was evaluated. Briefly, at the end of the cycle, 30 heads of lettuce for each treatment (N=90) were placed in a polyethylene bag in a refrigerator at +4°C. After twelve days, the condition of the heads was detected by quantifying the level of leaf rot using the 0 to 5 scale described above.

### 2.4 Statistical analysis

Statistical analyses were conducted to evaluate the effects of experimental treatments on various parameters related to crop yield, pest and disease incidence, and shelf life. A one-way analysis of variance (ANOVA) was performed to assess the impact of treatments on specific variables for each crop, including leaf mass, root mass, slug damage, and leaf rot during shelf life for lettuce; leaf mass, root mass, leaf miner damage, *Cercospora* leaf spot, Mn deficiency, and leaf chewing damage for chard; and bulb mass, bulb diameter, and root mass for onion. The significance of treatment effects was determined at a confidence level of P<0.05, and when significant differences were observed, a *post-hoc* Tukey’s Honest Significant Difference (HSD) test was applied to identify pairwise differences among treatments. Data was checked for normality and homogeneity of variances prior to conducting ANOVA. All statistical analyses were performed using STATISTICA software (version 14.0.1, StatSoft), and results were expressed as mean values with standard deviations or standard errors, as appropriate.

## 3 Results

### 3.1 Solid feedstock and compost tea chemistry

The organic carbon content was 27.3% and the nitrogen content was 2.3%, consequently the C/N ratio was 11.8. The feedstock was found to be sub-acid (pH 6.8) and with low salinity, not phytotoxic but with high content of humic and fulvic acids (**Table 1**). From a molecular point of view, the feedstock spectra are dominated by the O-alkyl-C region (61–90 ppm) and di-O-alkyl-C region (91–110 ppm), which are largely associated with sugars and polysaccharides like cellulose. The aliphatic alkyl-C region (0–45 ppm), characteristic of lipid like waxes, cutins and microbial products represents 19.5% of the spectrum (**Table 1**). The two aromatic fractions (111–140 and 141–160 ppm) together account for 12.8% of the spectra reflecting the presence of lignin and phenolic compounds. Finally, the carbonyl C fraction (161–190 ppm) and methoxyl C region (46–60 ppm) are also well represented and associate with N rich compounds.

**Table 1:** Content of organic carbon, total nitrogen, pH, electric conductivity, C/N ratio, humic and fulvic acids content, and ^13^C CPMAS NMR data of the solid fraction used to produce compost tea. Germination index values ​​>70 indicate the absence of phytotoxicity.

Parameters	Organic feedstock from G-Agro
Chemical	
C %	27.3
N %	2.3
C/N ratio	11.8
pH	6.8
EC dS/m	12.4
Humic and fulvic acids (%)	8.2
Water content (%)	13.5
Germination index	113
^13^C-CPMAS NMR	
Carbonyl-C (161–190 ppm)	7.7
O-subst. aromatic C (141–160 ppm)	6.4
H-C subst. aromatic C (111–140 ppm)	6.4
di-*O*-alkyl C (91–110 ppm)	9.6
*O*-alkyl C (61–90 ppm)	39.1
Methoxyl C (46–60 ppm)	11.2
Alkyl C (0–45 ppm)	19.5

Compost tea has a sub-acid pH, a high dissolved organic carbon concentration and consequently a very high BOD5, demonstrating the labile nature of the molecules dissolved in water. The *L. sativum* bioassay demonstrated absence of phytotoxicity for compost tea (**Table 2**). On the contrary, the nitrogen concentration in mineral forms is relatively low, as is the case for phosphorus and iron. The concentration of dissolved oxygen was of 4.6 mg L, a value in line with, although slightly lower than, that reported in the literature for aerobic compost teas. Finally, chloride and EC fall within the tolerance range of most horticultural species (**Table 2**). Microbiological analyses also excluded the presence of *E. coli* and *Salmonella* spp. in the solid feedstock.

**Table 2:** Chemical characteristics of compost tea immediately before the application. Germination index values ​​>70 indicate the absence of phytotoxicity.

Parameters	Organic feedstock from GW Agro
pH	6.4
EC (µS cm^-1^)	1370
DOC (mg l^-1^)	5123
BOD5 (mg l^-1^)	1350
Germination index	108
Dissolved oxygen (mg l^-1^)	4.6
Nitrate (mg l^-1^)	1.4
Ammonia (mg l^-1^)	7.2
Total Iron (mg l^-1^)	2.3
Sulphates (mg l^-1^)	170
Chlorines (mg l^-1^)	298
Phoshates (mg l^-1^)	18.4

### 3.2 Compost tea microbiome

**Figure 1:**
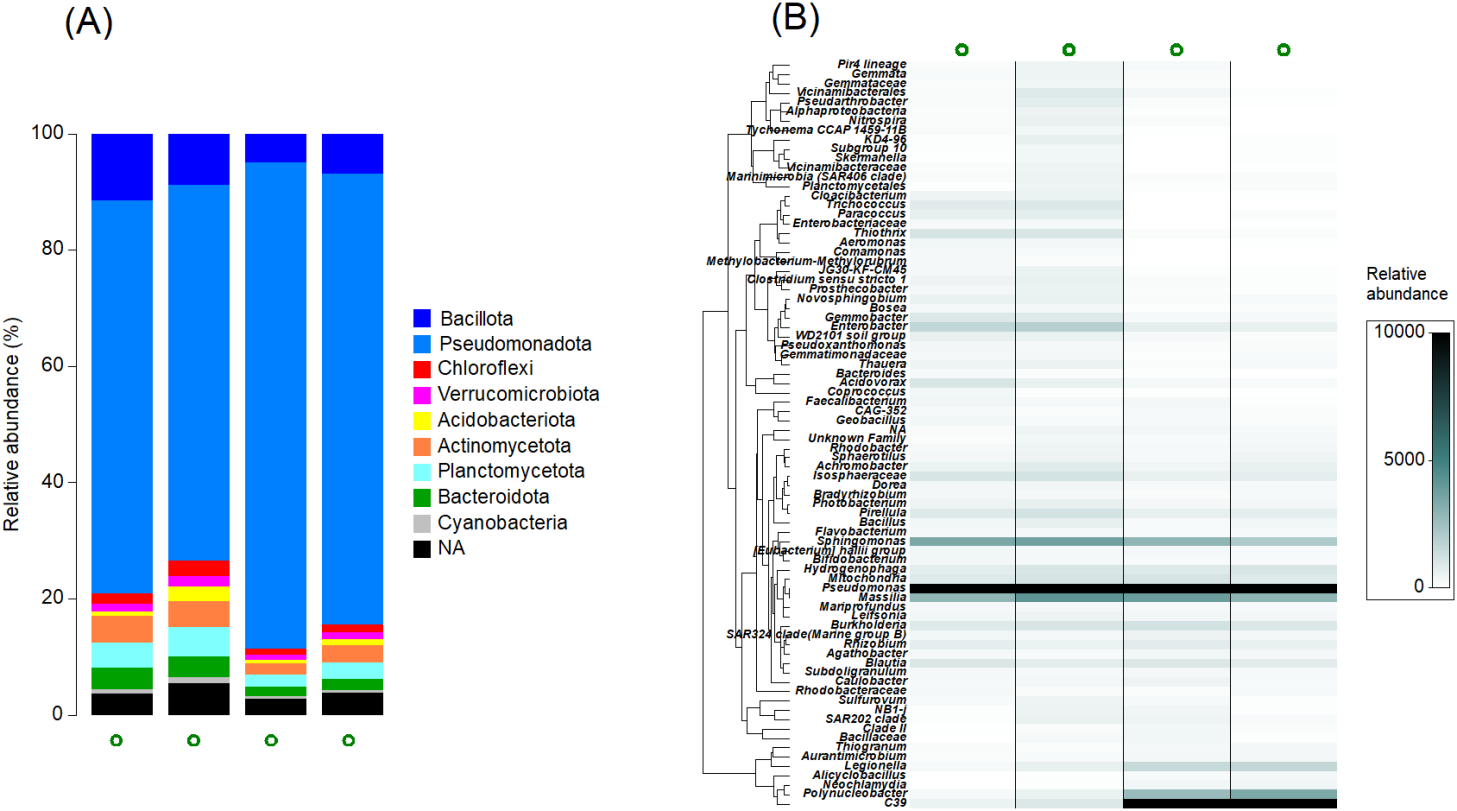
**(A)** The relative abundance of bacterial phyla for each replicate of the compost tea produced in four distinct brewing processes. **(B)** Heatmap showing relative abundance of the 100 most frequent Amplicon Sequence Variants (ASVs) in the bacterial community for each brewing of compost tea. The hierarchical clustering of variables is based on Whittaker’s association index.

For the four compost tea samples investigated, the DNA concentration was 90.9, 115.6, 107.2 and 125.2 ng μl^-1^, while the A 260/280 ratio was found to be suitable as it was greater than 1.8 (i.e. 1.84, 1.87, 1.88 and 1.84 for the four samples). Taxonomic classification of the bacterial community revealed that all compost tea replicates were predominantly composed of *Pseudomonadota*, followed by *Bacillota*, *Planctomycetota, Actinomycetota*, and *Bacteroidota*. In contrast, *Acidobacteriota, Cyanobacteria*, and *Verrucomicrobiota* were the least represented taxa (**Figure 1A**). At the genus level (**Figure 1B**), *Pseudomonas* emerged as the most abundant, comprising over 25% of the entire community, with *Massilla, Sphingomonas*, and *Bacillus* as other common genus.

### 3.3 Crop yield

**Figure 2:**
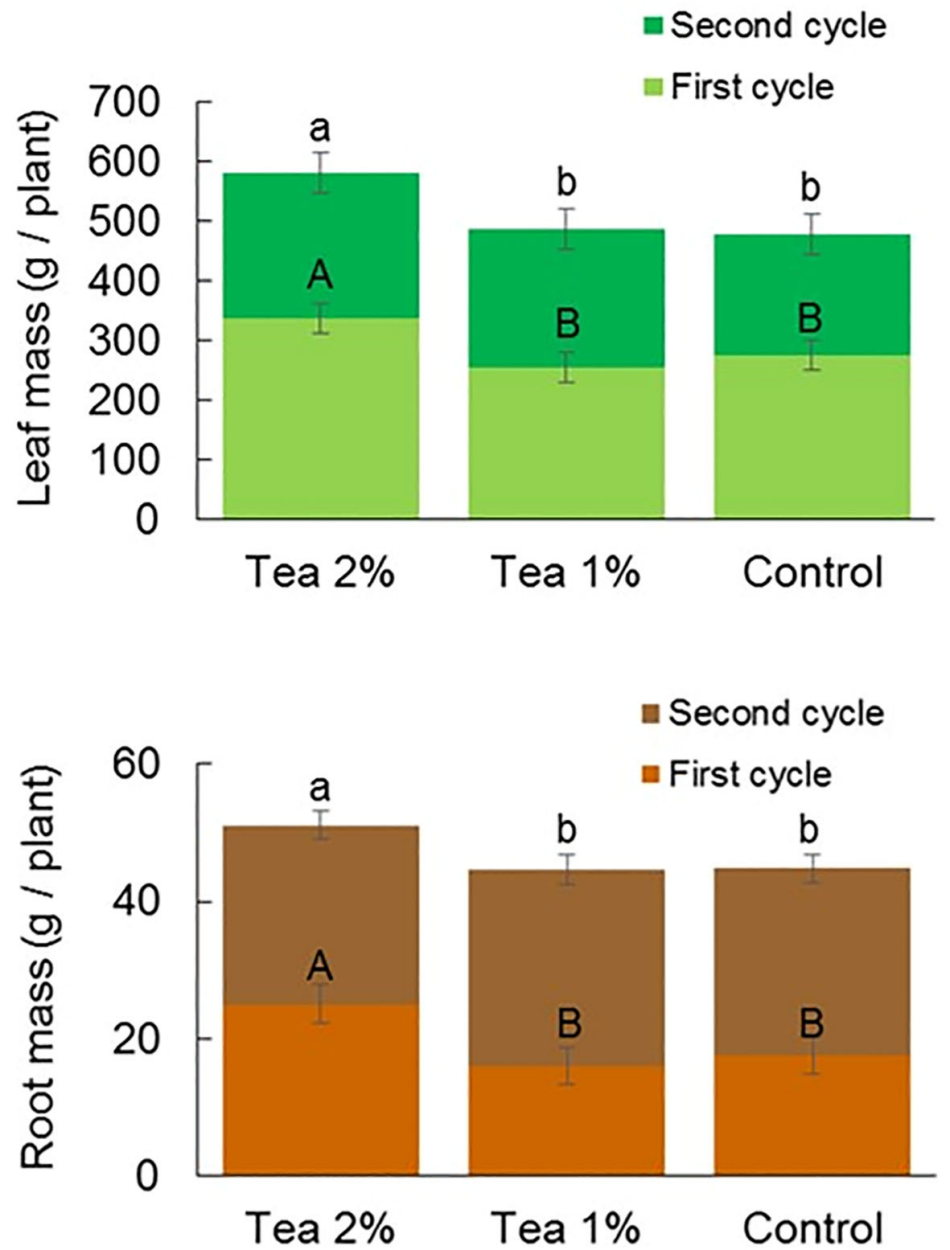
Above-ground (upper panel) and below-ground (lower panel) biomass of lettuce at the end of the first and second cultivation cycle without and with the application of compost tea at two concentrations. Different letter (lowercase letter = first cycle; uppercase letter = second cycle) indicate statistically significant differences (ANOVA Tukey test; *P* < 0.05).

The leaf biomass of lettuce was higher in the 2% compost tea treatment compared to the control in both the first and second cultivation cycle (**Figure 2**). Also, in the case of root biomass, the application of 2% compost tea caused an increase in biomass compared to the untreated control. In the case of 1% compost tea, no differences were detected compared to the control in both the first and second cultivation cycle.

**Figure 3:**
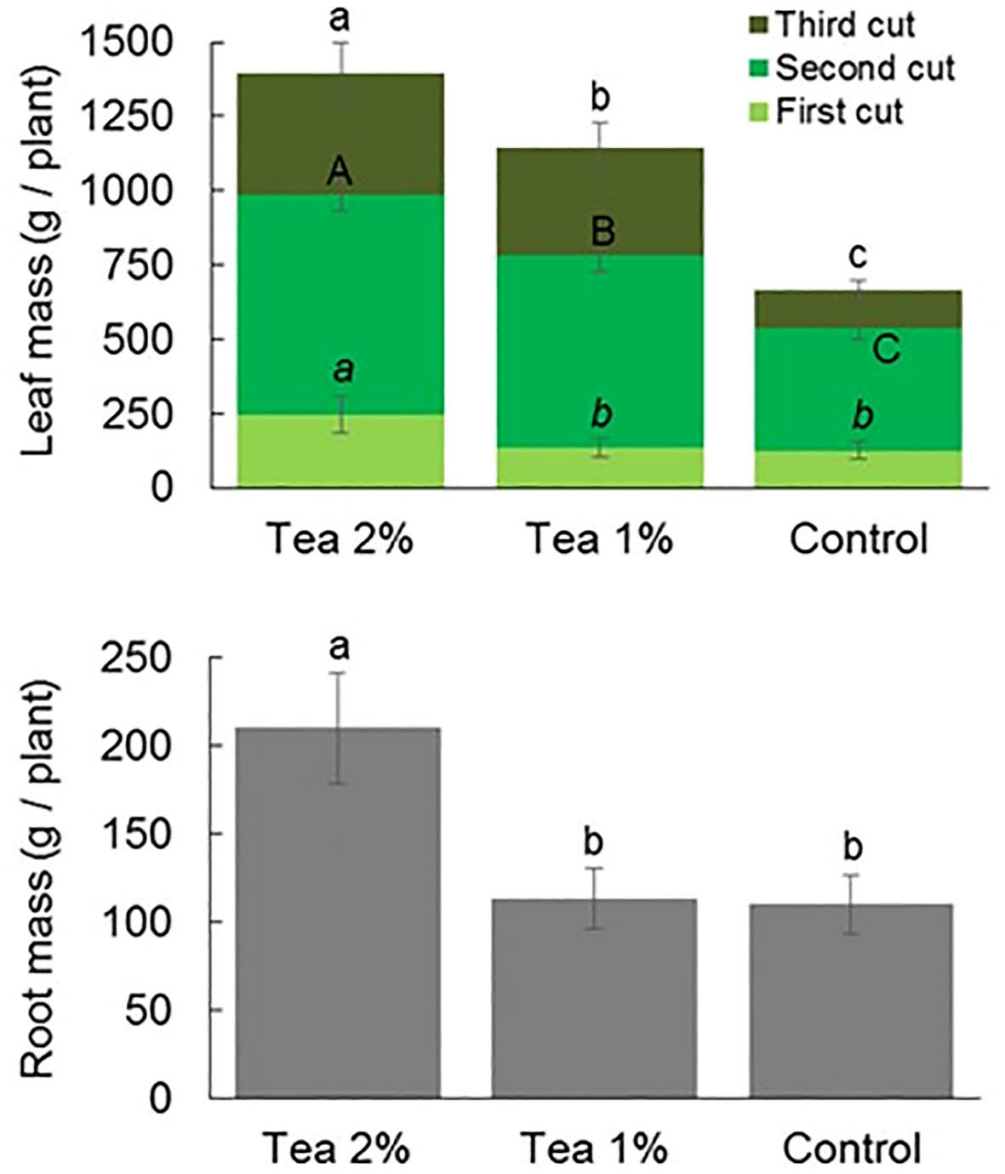
Above-ground (upper panel) and below-ground (lower panel) biomass of chard at the end of three consecutive leaf cut without and with the application of compost tea at two concentrations. Different letter (lowercase letter = first cut; uppercase letter = second cut; lowercase italic letter = third cycle) indicate statistically significant differences (ANOVA Tukey test; *P* < 0.05).

Regarding chard, the weekly application of 2% compost tea caused an increase in leaf biomass in all three cuts as well as in root biomass at the end of the cycle (**Figure 3**). In the case of 1% compost tea, root biomass was not different from the control while leaf biomass was higher in the second and third cuts but not in the first. Overall, the application of 1% and 2% compost tea caused an increase in leaf biomass of 72% and 110% compared to the control, respectively.

**Figure 4:**
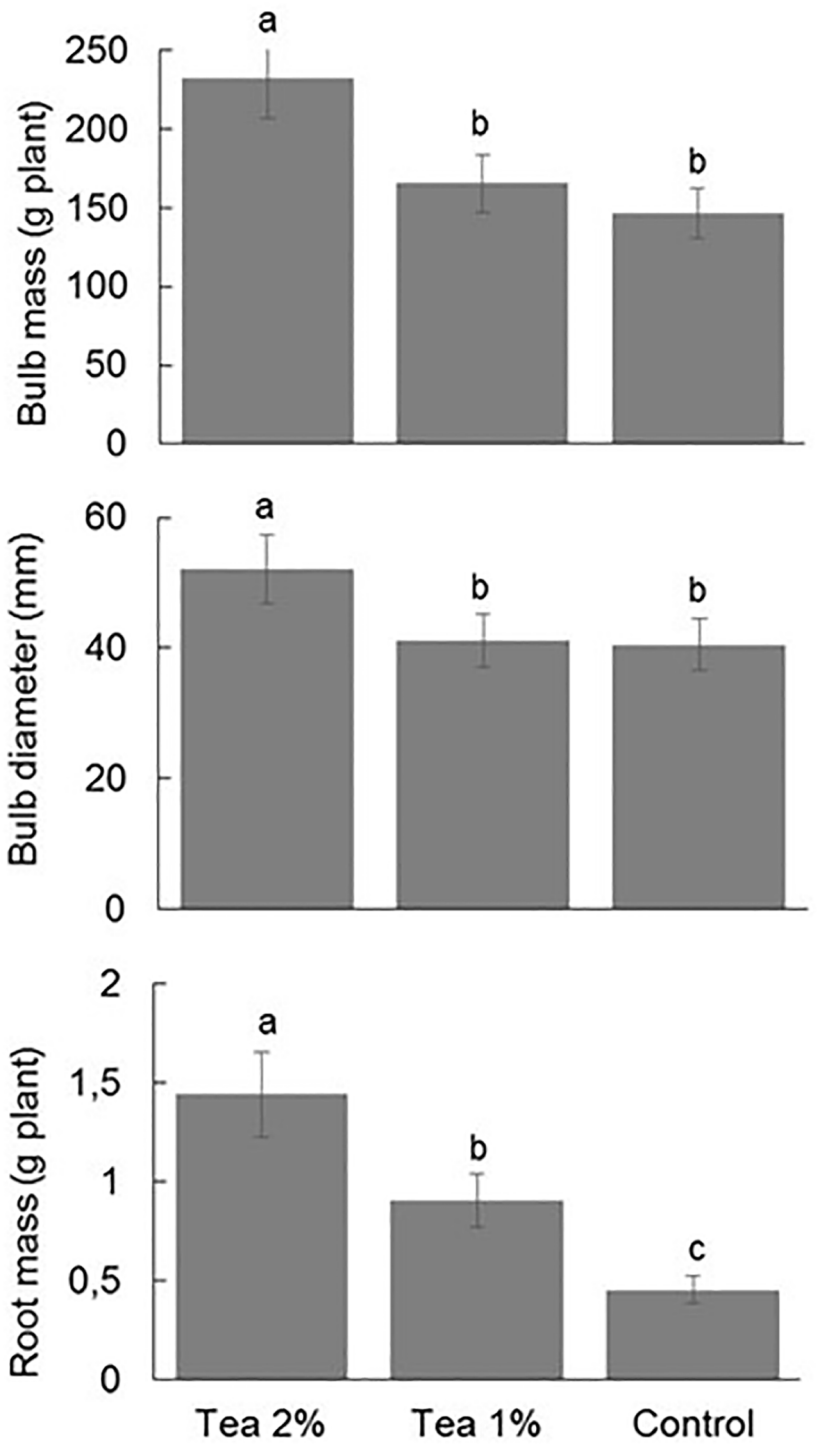
Bulb mass (upper panel), bulb diameter (middle panel) and root biomass (lower panel) of onion at the end of three consecutive leaf cut without and with the application of compost tea at two concentrations. Different letters indicate statistically significant differences (ANOVA Tukey test; *P* < 0.05).

In the case of onion, the 2% compost tea increased all the biometric parameters evaluated, i.e. bulb diameter, bulb mass and root biomass (**Figure 4**). The 1% compost tea instead caused a much less marked increase in bulb and root mass, while no differences were detected compared to the control for bulb diameter.

### 3.4 Lettuce shelf life and chard pests and disease

**Figure 5:**
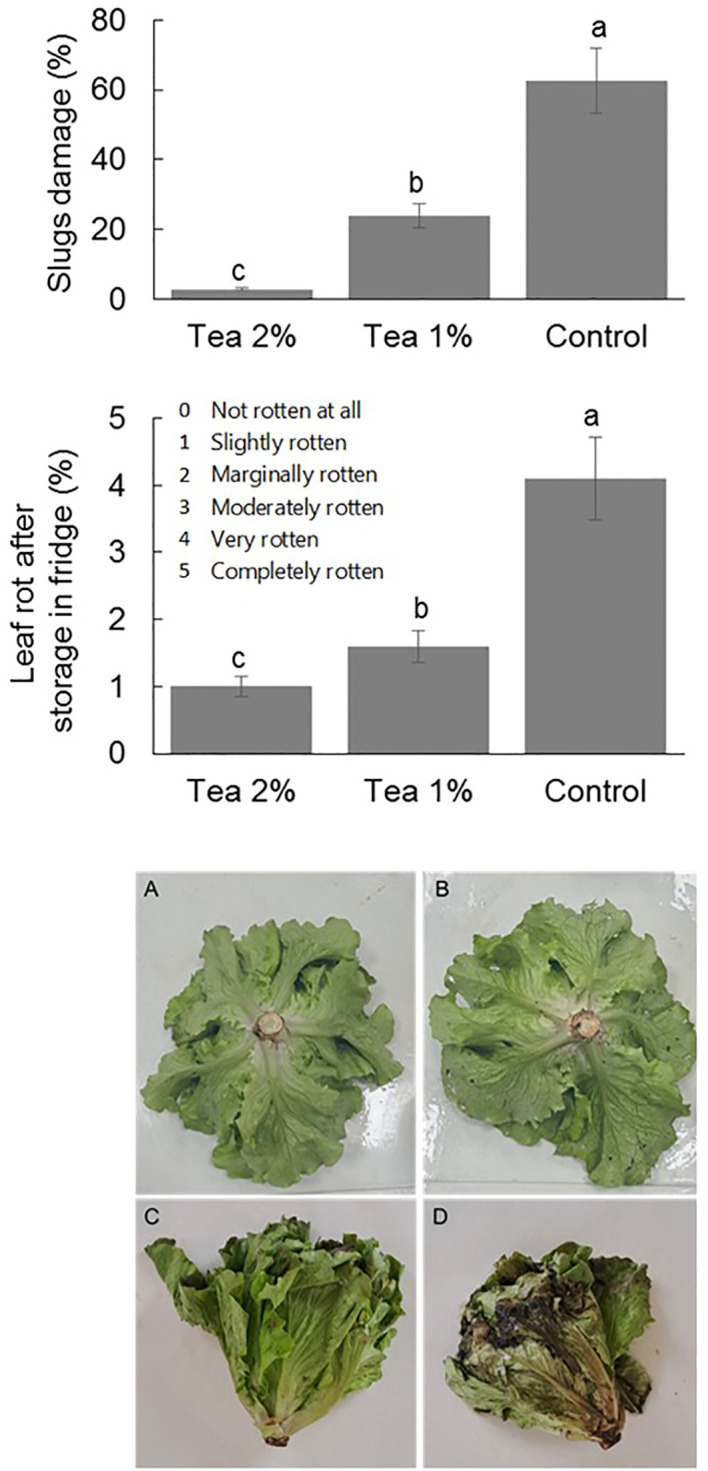
Slugs damage (upper panel) and leaf rot after twelve days of fridge conservation (middle panel) of lettuce at the end of second cycle without and with the application of compost tea at two concentrations. Different letters indicate statistically significant differences (ANOVA Tukey test; *P* < 0,05). Pictures shows the damage caused by snails in the 2% compost tea **(A)**, in the control **(B)**, and the leaf rot after one week of storing the lettuce in the refrigerator at the end of the second cycle with the application of compost tea at 2% **(C)**, and without application **(D)**.

During the second production cycle of lettuce an attack of slugs was observed, especially on the external leaves and the basal leaves of the head (**Figure 5**). The application of 2% compost tea caused a drastic reduction in the incidence of slugs, an effect that was highly significant also in the case of 1% compost tea compared to the control.

As regards the experiment of refrigerator storage of lettuce heads, it was possible to find that compost tea at both concentrations significantly improved the shelf life. In fact, the fraction of rotten leaves after twelve days of storage was significantly higher in control compared to the heads treated in the field with compost tea (**Figure 5**).

**Figure 6:**
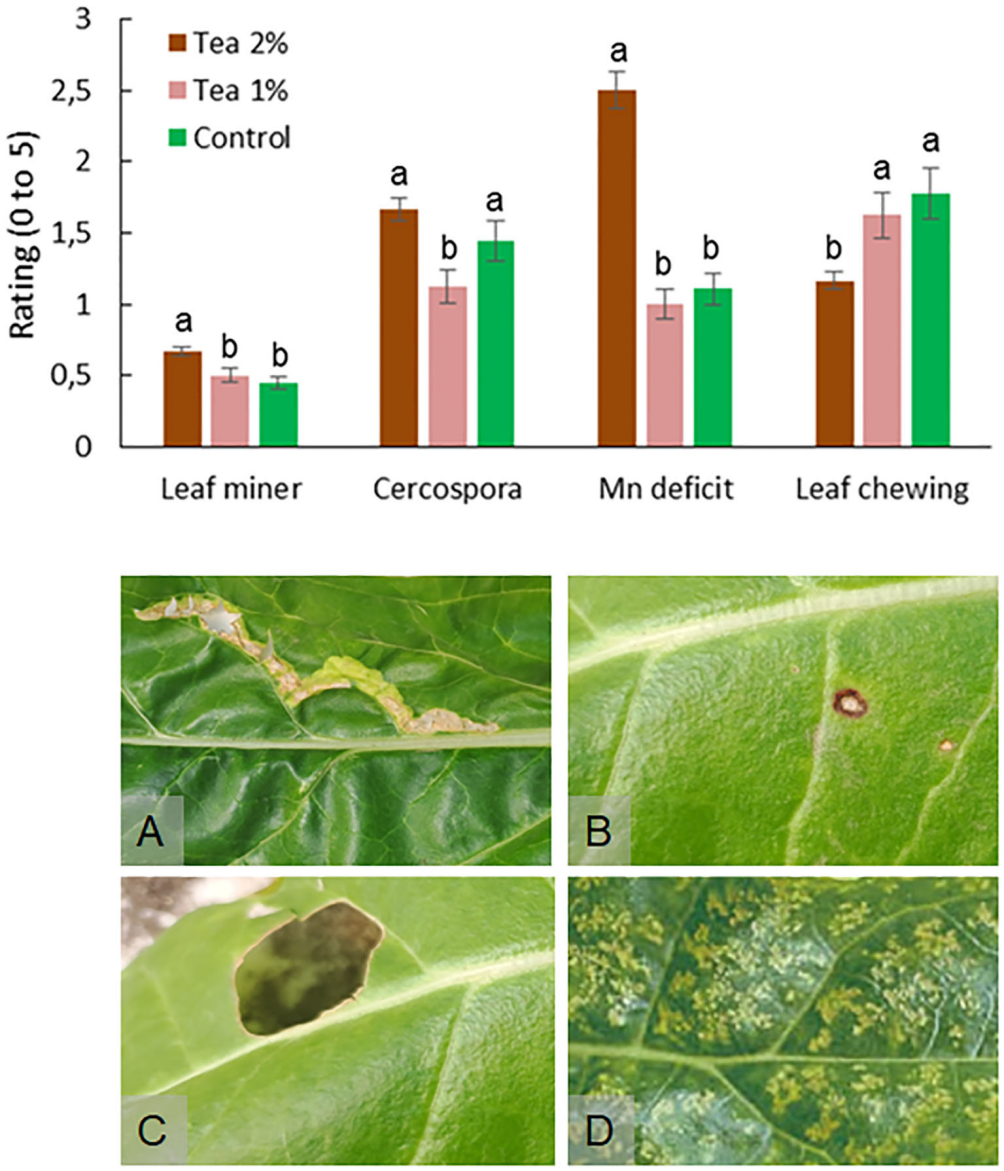
Impact of leaf miner, cercospora leaf spot, manganese (Mn) deficit and leaf chewing insects on leaf of chard at the end of third cut without and with the application of compost tea at two concentrations. Different letters indicate statistically significant differences (ANOVA Tukey test; *P* < 0.05). Pictures shows examples of leaf attacked by leaf miner **(A)**, cercospora leaf spot **(B)**, leaf chewing **(C)**, and Mn deficiency **(D)**.

In the case of chard, the application of compost tea recorded contrasting effects on the various phytopathological problems. In fact, 2% compost tea compared to the control caused a slight but significant increase of leaf-mining insects in addition to an increase in the incidence of Mn deficiency (**Figure 6**). On the contrary, compost tea 2% determined a reduction in the attack of chewing insects and no effect on the incidence of cercospora leaf spot. Compost tea 1%, instead, did not detect any differences compared to the control except for a slight reduction in the incidence of cercospora leaf spot. Finally, during the onion cultivation no phytopathological problems were detected.

## 4 Discussion

### 4.1 Feedstock chemistry and compost tea microbiome

The starting feedstock used is mainly made up of tissues of vegetal origin such as straw of grasses and nitrogen-fixing hay. In fact, the profile returned by the ^13^C CPMAS NMR analysis is similar to that of previously described vegetal litters (Bonanomi et al., 2024) with a prevalence of the O-alkyl-C and di-O-alkyl-C region fractions which are typical of sugars and polysaccharides like cellulose and hemicellulose (Kögel-Knabner, 2002). Furthermore, the profile shows a significant presence of the aliphatic fraction (alkyl-C region), characteristic of lipid like waxes and cutins. Microbial by-products linked to the mature manure fraction that has been reworked by bacterial and fungal activity also contribute to this region. The analysis also revealed the presence of a substantial aromatic fraction (111–140 and 141–160 ppm) which indicates that the vegetal tissues contained lignin. Finally, the carbonyl C fraction (161–190 ppm) and methoxyl C region (46–60 ppm) associated with N rich compounds are probably related to tissues of nitrogen-fixing species (Bonanomi et al., 2024). During the preparation of compost tea, materials rich in labile carbon and simple sugars such as molasses, a material often used to promote microbial growth during brewing, were not added (Diacono and Montemurro, 2011; Bethe et al., 2017). The use of highly fermentable substrates, on the one hand, promotes rapid microbial growth, but on the other hand, causes an equally rapid consumption of oxygen with the risk of inducing anaerobic conditions as recently demonstrated (Ou-Zine et al., 2022). It is well established that aeration during the brewing process is the most important ecological factor, together with the chemical composition of the feedstock, in determining the composition of the microbiome (Scheuerell and Mahaffee, 2002). Consequently, anaerobic conditions should be avoided because they lead to the development of bad odors generated during fermentation with the formation of aldehydes and short-chain fatty acids (Wang et al., 2023), and because they increase the risk of development of human pathogens such as Enterobacteriaceae, *Escherichia coli* and *Listeria monocytogenes* (Palmer et al., 2010). Microbiome analyses of our compost tea have detected the presence of bacteria belonging to the genera *Legionella* and *Enterobacter*. Although the resolution of the methodology used does not allow assigning these ASV to human pathogenic species, the relative abundance of these groups suggests a potential contamination during the brewing phases since the parental feedstocks are free of human pathogens. This aspect is particularly relevant for the food security of crops such as lettuce that are consumed fresh. Future studies will have to ascertain the presence of human pathogens not only in the feedstock but also in the product following the brewing process and, if necessary, modify the production process to reduce their abundance.

To ensure consistent fermentation and avoid anomalies, the substrate used in this study was carefully balanced. It consisted of both a labile fraction and a more stable, microbially processed material, specifically mature manure, along with plant tissues that varied in composition, some being rich in lignin while others were abundant in nitrogen. This balanced chemical composition played a crucial role in shaping the bacterial microbiome, which, was primarily dominated by microbial groups within the *Pseudomonadota* phylum, such as *Pseudomonas, Massilla*, and *Sphingomonas*. Interestingly, the most abundant microbial groups are known for their ability to stimulate plant growth as well as for their suppressive action against plant pathogens. For example, strains of *Pseudomonas*, the most abundant group in our compost tea, are known for suppression against *Rhizoctonia solani* (Mazzola and Gu, 2002), but also take-all disease of wheat (Weller et al., 2002). *Pseudomonas*, however, is a very large genus that includes, in addition to beneficial species, also numerous plant and human pathogens such as *P. aeruginosa*. Future studies will have to identify the *Pseudomonas* species present in compost tea to better understand their functions and role in the observed stimulation. The genera *Massilia* and *Sphingomonas* are certainly less studied than *Pseudomonas*, but some research works have revealed a rather association between disease suppression and the relative abundance of such groups (Mendes et al., 2011). For example, Bonanomi et al. (2018) found that the relative abundance of *Massilia* and *Sphingomonas* was higher in organic farms, compared to conventional one, with positive impact on crop performances and suppression of *R. solani*. The compost tea microbiome, although dominated by *Pseudomonadota*, also presents, in all the analyzed batches, an abundant presence of *Bacillus* belonging to the Bacillota phylum. *Bacillus* is also a very large genus, but some studies based on plate counts have detected their abundant presence in compost tea (Kim et al., 2015). Furthermore, other studies have shown that suppressive composts are particularly rich in *Bacillus* (Pane et al., 2022), suggesting that this microbial group could also play an important role in the investigated compost tea. It is important to note the limitations of the study which investigated only the bacterial fraction of the microbiome, while future studies will have to extend the investigations to other components such as protozoa, nematodes and fungi.

One of the key findings of this study is the remarkable uniformity of the microbiome across four distinct brewing processes. The inconsistency and variability in compost tea have long been cited as major drawbacks, leading to scepticism about its efficacy, with some critics dismissing it as an “esoteric” practice or likening it to “snake oil remedies” (Gillman, 2008). However, our results challenge this perception by demonstrating that starting with a chemically balanced feedstock and standardizing critical process conditions, such as brewing duration and oxygenation, can yield a compost tea with a relatively consistent and stable microbiome. This finding underscores the importance of controlling input materials and process parameters to produce a more reliable and reproducible product.

### 4.2 Vegetables yield

Weekly application of compost tea, especially at the higher concentration of 2%, promoted the growth of three plant species belonging to three different botanical families i.e. Chenopodiaceae, Asteraceae and Liliaceae, suggesting a broad spectrum of action. Growth promotion was highest for chard leaf production (+110%), intermediate for onion bulb biomass (+58%) and lower, but still significant, for lettuce (+21%). Biostimulant effects on the growth of horticultural species, in many cases even very significant and higher than those found in our study, are often reported in the literature (Scheuerell and Mahaffee, 2002). A recent meta-analysis (Curadelli et al., 2023) reported a growth promotion of species treated with compost tea compared to the control of +92%, although the sample size was small. Previous studies have shown that both lettuce (Kim et al., 2015; Pane et al., 2014) and onion (Orden et al., 2021) respond positively to compost tea, although the products used, and the application methods are different. In our study, the response of plants was concentration dependent, with the best results obtained at the highest concentration. This result is not surprising since the dosage used for the production of compost tea is much lower, with a ratio of organic feedstocks to water of 1:50, compared to what reported in the literature where the commonly used range varies from 1:5 to 1:10 (Pilla et al., 2023). In our case, a lower dosage, appropriately balanced by a better quality of the starting organic feedstock and by controlled process conditions, allows on the one hand a saving of dry mass for the farmer, a greater ease in carrying out the brewing and, with a smaller quantity of organic matter in suspension, the risk of blockage of the pipes and nozzles used for the application of the product is reduced (Idbella et al., 2023).

Considering the low dosage used, equivalent to 20 g of organic feedstock per L, and the relative low content of dissolved organic carbon and dissolved nutrients, the notable biostimulant effect observed is not immediately explainable. In fact, the nutritional contribution for N and P, considering their average concentration in compost tea (**Table 2**), and the application rate per treatment (1600 l ha**^-1^**) can be estimated in only 13.2 for mineral N forms and 22.2 for P in g^-1^ ha^-1^ treatment^-1^. It is evident that the pure nutritional action can play a role but cannot fully explain the observed effects, especially compared to the contributions of dissolved organic carbon which amounts to approximately 7989.8 g^-1^ ha^-1^ treatment^-1^. The biostimulant effect could be attributed to organic molecules such as humic and fulvic acids (Pane et al., 2016), molecules with auxin-like action (González-Hernández et al., 2022), but also to the presence of heterologous DNA (Lanzotti et al., 2022) up to the direct action of the microbiome (St. Martin et al., 2020). In relation to this last point, the compost tea studied was dominated by bacteria of the genera *Pseudomonas, Sphingomomas, Bacillus* as well as *Massilla*, all genera rich in species with beneficial action and often reported as plant growth promoting rhizobacteria (Bonanomi et al., 2022; Wagstaff et al., 2010). Our study was not designed to unravel the mechanisms underlying the beneficial action of compost tea, but future studies may evaluate the relative contribution of organic molecules and microbiome by comparing compost tea as is with the same product filter-sterilized so as not to alter its chemical composition.

### 4.3 Pest, disease and lettuce shelf life

Lettuce is one of the most consumed leafy vegetables in the world, but it is also one of the most delicate with significant losses in the field and in post-harvest (Wagstaff et al., 2010). Our study has given the first indication that the use of compost tea can be useful for the control of slugs and for the extension of shelf life when stored in the refrigerator. With respect to the first point, slugs can cause significant losses also from a qualitative point of view with the release of their feces, especially in organic agriculture where few control tools are available such as marsh flies, carabid beetles and parasitic nematodes (Hass et al., 1999; Barua et al., 2021). Slugs are also a problem in conventional agriculture where the few registered products based on metaldehyde are subject to restrictions for environmental toxicity and toward non-target organisms. Our study, for the first time, suggests that compost tea can considerably reduce the incidence of these molluscs. The effect could be caused by a repellent action or by an induction of resistance that modifies their palatability. Further studies are needed to confirm this action through experiments in controlled conditions where slug density must be included as a manipulated factor. In relation to shelf life, plants treated in the field with compost tea showed, after twelve days in the refrigerator, a lower browning and rotting of the leaves. The shelf life of lettuce, considering its economic importance, has been studied extensively and is linked to genetic varietal factors as well as cultivation and storage conditions (Peng and Simko, 2023). Among the agronomic factors, previous studies have shown that shelf life improves by avoiding excess nitrogen fertilization (Tsouvaltzis et al., 2020), while the use of biostimulant products based on humic acids or natural extracts can have beneficial effects (Canellas et al., 2015). This is the first study that reports a substantial improvement in shelf life through applications in the field, and not in post-harvest, of compost tea. This effect could be linked to a different type of nitrogen nutrition, less based on nitrate nitrogen, to a change in the structure and size of the cells, or to an accumulation of substances with antioxidant action such as phenols as recently reported by a study that applied compost tea in hydroponic cultivations (Hartadiyati and Rusilowati, 2020). Future studies will have to confirm the positive effect on shelf life on the one hand and investigate the risks of contamination with human pathogenic species on the other.

Unlike onion that did not show any problems, chard showed several problems of both biotic and abiotic nature. The application of compost tea in this case had variable effects both positive and negative. In the case of insects, the effect was a slight decrease for chewing organisms but a slight increase for leaf miners. Metabolomic insights will be necessary to understand how compost tea modifies the attractiveness of leaf tissue toward insects with different ecology. Regarding fungal pathogens, cercospora leaf spots are the main adversity for chard (Rangel et al., 2020). In our study, based on natural inoculum present in the greenhouse, the incidence and severity were relatively low with no differences between compost tea 2% and control. Previous studies have shown that compost tea can suppress both foliar pathogens such as powdery mildew, gray mold (*Botrytis cinerea*), *Alternaria alternata*, and numerous soilborne pathogens (Pilla et al., 2023; Kim et al., 2015; Pane et al., 2012). Future studies could further evaluate the impact of this product on an important pathogen such as *Cercospora beticola*. Finally, during cultivation a widespread interveinal chlorosis attributable to Mn deficiency was detected. This microelement can be deficient for Chenopodiaceae especially in some soils, such as the one used in our study and because it is not normally integrated through the normal fertilization practice. In our study the incidence of Mn deficiency was higher in the plots treated with 2% compost tea. This could be linked to a greater need for the microelement due to the greater growth of the crop. The result has important application implications since compost tea is naturally deficient in Mn, suggesting the possibility of integrating the product with the addition of this microelement and to be used in specific contexts when such a deficiency can occur.

## 5 Conclusions

The compost tea studied, based on a mixture of plant species, straw and mature manure, was particularly promising in promoting the development of lettuce, onion and chard. In addition, early evidence suggests that weekly application may be useful for the control of some harmful organisms such as slugs or in prolonging the shelf life of lettuce. The compost tea studied, moreover, is characterized by a microbiome dominated by *Pseudomonadota*. This microbiome with overall beneficial effects can be generated, with good uniformity, using a homogeneous starting organic feedstock and respecting the process conditions in terms of oxygenation, matrix:water ratio and brewing duration. Respecting the process conditions also allows a reduction in the dosage i.e. ratio 1:50 between organic matrix and water, and a reduction in the brewing duration to 48 hours compared to values that can reach seven and even ten or fourteen days in literature (Pilla et al., 2023). The reduction of dosage and the shortening of the brewing phase, without affecting its functionality, are aspects of great practical relevance that could favor the popularity of this low environmental impact strategy. An aspect not investigated by our study is the impact of the dosage on the biostimulant effects, a factor that will have to be studied to evaluate whether compost tea is effective even by reducing the application rate. Future studies of our research group are aimed at investigating the suppressive effects toward foliar and root pathogens as well as toward the control of harmful insects. Finally, a critical aspect that limits the spread of compost tea use is the need to produce it on the farm and apply it in a timely manner without the possibility of storage. In fact, the effectiveness of compost tea is strongly linked to an active microbial community capable of performing its functions in the rhizosphere and phyllosphere. Unlike beneficial microorganisms sold in containers and which can be stored for months, compost tea must be produced at the moment of use. This is undoubtedly a practical disadvantage but one that is reflected, with an inevitable trade-off, in greater effectiveness. Future studies may evaluate the possibility of storing compost tea for short periods of time on the order of days or weeks.

## Data Availability

The original contributions presented in the study are included in the article/**Supplementary Material**. Further inquiries can be directed to the corresponding author.
